# Review of Defective NADPH Oxidase Activity and Myeloperoxidase Release in Neutrophils From Patients With Cirrhosis

**DOI:** 10.3389/fimmu.2019.01044

**Published:** 2019-05-08

**Authors:** Richard Moreau, Axel Périanin, Vicente Arroyo

**Affiliations:** ^1^Inserm, U1149, Centre de Recherche sur l'Inflammation, Paris, France; ^2^UMRS1149, Université Paris Diderot-Paris 7, Paris, France; ^3^Département Hospitalo-Universitaire UNITY, Service d'Hépatologie, Hôpital Beaujon, Assistance Publique-Hôpitaux de Paris, Clichy, France; ^4^Centre National de la Recherche Scientifique (CNRS), Paris, France; ^5^EF Clif, EASL-CLIF Consortium and Grifols Chair, Barcelona, Spain

**Keywords:** liver disease, infection, neutrophil dysfunction, granule exocytosis, serine-threonine protein kinases

## Abstract

Patients with decompensated cirrhosis are highly susceptible to develop bacterial infections and these can trigger multiorgan failure associated with high in-hospital mortality. Neutrophils from patients with decompensated cirrhosis exhibit marked alterations that may explain the susceptibility of these patients to develop bacterial infections. These neutrophil alterations include marked defects in intracellular signaling pathways involving serine/threonine kinases such as protein kinase B (AKT), p38-mitogen-activated protein kinase (MAPK), and the MAP kinases1/2; activation of the NADPH oxidase complex; myeloperoxidase (MPO) release; and bactericidal activity of neutrophils stimulated by the bacterial peptide formyl-Methionine-Leucine-Phenylalanine (fMLF). Impaired activity of the NADPH oxidase 2 (NOX2) complex is also related to reduced levels of expression of its major components through post-transcriptional mechanisms. In addition, the catalytic NOX2 component gp91^*phox*^ is subject to degradation by elastase highly present in patients' plasma. A defect in the protein kinase B (AKT) and p38 MAPK-mediated signaling pathways may explain the decrease in phosphorylation of p47^*phox*^ (an important component of the NADPH oxidase complex) and MPO release, in response to neutrophil stimulation by fMLF. Most of these alterations are reversible *ex vivo* with TLR7/8 agonists (CL097, R848), raising the possibility that these agonists might be used in the future to restore neutrophil antibacterial functions in patients with cirrhosis.

## Introduction

The natural history of cirrhosis, the most common chronic liver disease, is characterized by episodes of acute decompensation (e.g., development of ascites, gastrointestinal hemorrhage, or hepatic encephalopathy) (1). Patients with acutely decompensated cirrhosis are usually admitted to the hospital (1). Of these, 70% have traditional acute decompensation do not exhibit any organ dysfunctions or failures and have a 28-day mortality rate of <5%. The 30% remaining patients have acute-on-chronic liver failure (ACLF) which is defined by the presence of organ failures and a 28-day mortality rate ranging from 20 to 80% or more, depending on the number of failing organs (1). Patients with cirrhosis are highly susceptible to develop acute bacterial infection, which is the most common trigger of traditional acute decompensation and ACLF (1, 2). Studies have shown that neutrophils from patients with cirrhosis exhibit *ex vivo* defective adenine dinucleotide phosphate (NADPH) oxidase 2 (NOX2) (3–5) and of myeloperoxidase (MPO) exocytosis (4), which both may contribute to the susceptibility to infection in patients with cirrhosis. Before summarizing our knowledge about the defective neutrophil functions in cirrhosis, it is important to have some general information on NADPH oxidase activity and MPO release in neutrophils.

## NADPH Oxidase Activation and MPO Release in Neutrophils From the General Population

Almost 90% of granulocytes in peripheral blood are composed of neutrophils which represent the first line of cellular defense against bacterial infections and play an important role in innate immunity and inflammation. Circulating neutrophils are the first to arrive at a site of infection, and they stay for only a short time (the first 24 h), most of them undergoing cell death in the inflamed tissue as a consequence of their antibacterial effector functions (6). Phagocytosis of bacteria at the infection site activates neutrophil functions, such as the release of proteases, bactericidal peptides and reactive oxygen species (ROS) (7, 8). ROS production is initiated by the generation of superoxide anion (${\text{O}}_{2}^{-.}$) by the NADPH oxidase. In the phagosomes, ${\text{O}}_{2}^{-.}$ reacts with protons to form hydrogen peroxide (H_2_O_2_), which is used by myeloperoxidase (MPO, an azurophilic [or primary] granule lumen protein) to produce the highly bactericidal ROS, hypochlorous acid. The rapid increase in oxygen and glucose consumption, together with ROS overproduction during neutrophil NADPH oxidase activation, is known as “respiratory burst” (RB). NADPH oxidase is a multicomponent protein (see below); an inherited defect in the expression of one of these components results in a rare disease called chronic granulomatous disease, which is characterized by a defect in ROS production in phagocytes and an increased susceptibility to recurrent bacterial and fungal infections (7). On the other hand, excessive neutrophil ROS production can cause tissue damage (7, 8). The importance of effective MPO release is highlighted by the findings in *MPO*-knockout mice of increased prevalence of infections, prolonged inflammation, and shorter survival (9, 10).

### NADPH Oxidase Activity

In its active state the NADPH oxidase is a multiprotein complex comprising the catalytic core flavocytochrome *b*_558_ heterodimer consisting in two associated transmembrane proteins, gp91^*phox*^ (i.e., cytochrome b-245 heavy chain, commonly called NOX2) and p22^*phox*^ (i.e., cytochrome b-245 light chain), and four proteins recruited from the cytosol, including p67^*phox*^ (i.e., neutrophil cytosol factor 2), p47^*phox*^ (i.e., neutrophil cytosol factor 1), p40^*phox*^ (i.e., neutrophil cytosol factor 4), and Rac2 (7). The oxidase is fully activated when cytosolic and membrane proteins are assembled into a complex, which makes gp91^*phox*^ able to use cytosolic NADPH to produce ${\text{O}}_{2}^{-.}$ (7, 8, 11).

**Figure 1:**
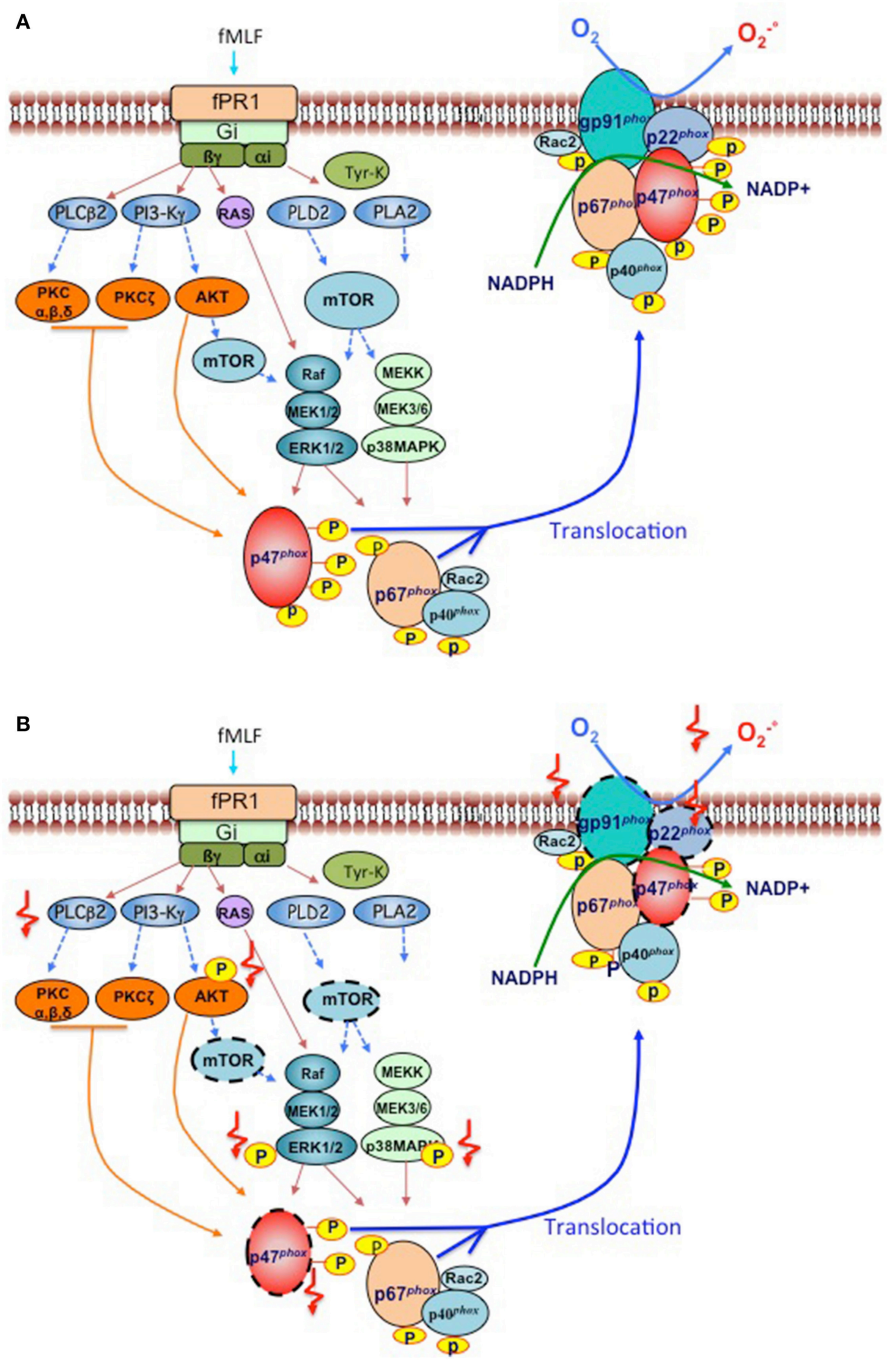
Signaling pathways involved in phosphorylation and activation of the NADPH oxidase induced by bacterial peptides in human neutrophils from respectively “healthy subjects” and “cirrhotic patients”. **(A)** Healthy subjects. The binding of the bacterial formylated peptide fMet-leu-Phe (fMLF) to its Gi-protein-coupled receptor fPR1, triggers the activation of various major early signaling effectors such as phospholipase C (PLCß2), Phospholipase D (PLD2), Phospholipase A (PLA2), Phosphatidylinositol-4,5-bisphosphate 3-kinase (PI3-Kγ), tyrosine kinases, and the small G-protein Ras. Second messengers produced by phospholipases stimulate various protein kinases, such as protein kinase C (PKC) isoforms, protein kinase B (AKT1/2), mammalian target of rapamycin (mTOR), which in turn activates two major families of mitogen-activated protein kinases (MAPKs), including ERK1/2 and p38-MAPK. PKCs and MAPKs phosphorylate cytosolic components of the NADPH oxidase (p47^*phox*^, p67^*phox*^, p40^*phox*^) which allows their translocation to the plasma membrane, together with the small G protein Rac2, to activate a cytochrome b, constituted with the gp91^*phox*^ (also known as NOX2) and its partner p22^*phox*^. The activated NOX2 reduces oxygen to superoxide (${\text{O}}_{2}^{-.}$) at the expense of NADPH. **(B)** Patients with cirrhosis. Two types of deficiencies have been identified in neutrophils of cirrhotic patients stimulated *in vitro* by bacterial peptides; those which decrease the activation/phosphorylation of signaling effectors such as AKT, MAP-Kinase ERK1/2, and p38-MAP kinase, p47^*phox*^; and PLCβ2 activity (indicated by 
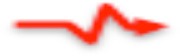
) and those which decrease the expression of protein effectors (indicated by a dashed outline, 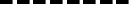) especially for mTOR, gp91^*phox*^, p22^*phox*^, and p47^*phox*^. These alterations lead to a deficient production of superoxide anion by gp91^*phox*^.

Different molecules can activate neutrophil NADPH oxidase including the bacterial peptide formyl-Met-Leu-Phe (fMLF), the complement fragment C5a, opsonized bacteria, opsonized zymosan and chemical agents such as calcium ionophores and the protein kinase C (PKC) activator, phorbol-myristate acetate (PMA) [reviewed in (11)]. FMLF, engages the surface formyl peptide receptor fPR1, a G-protein-coupled receptor, to activate several intracellular phospholipases, protein tyrosine kinases, serine/threonine kinases, including PKC isoforms, protein kinases B and B beta (hereafter called AKT1and AKT2, respectively), mammalian target of rapamycin (mTOR), and mitogen-activated protein kinases (MAPK), which include p38-MAPK and MAPK 1 (hereafter called ERK2) and MAPK 3 (hereafter called ERK1) (Figure 1A). Serine/threonine kinases phosphorylate the components of the NADPH oxidase (Figure 1A) at sites which are detailed in Table 1 and contribute to the assembly of the complex and ${\text{O}}_{2}^{-.}$ production. Of note, it has recently been shown that during the first hour of their *ex vivo* fMLF stimulation of neutrophils from healthy subjects, these cells release the protease elastase (contained in azurophil granules and specific [or secondary] granules) in the extracellular milieu to induce degradation of transmembrane gp91^*phox*^ (5). This degradation is followed by that of p22^*phox*^, which is an elastase-independent process, and might be a consequence of gp91^*phox*^ degradation that would render p22^*phox*^ unstable and degradable by intracellular proteases (5). The two cytosolic components of the NADPH oxidase complex, p47^*phox*^ and p40^*phox*^ are not affected by fMLF-induced elastase release (5).

**Table 1:** The different components of the NADPH complex, their site(s) of phosphorylation, phosphorylating serine/threonine kinases, and effects of phosphorylation on the NADPH complex (7, 8, 11).

**Gene symbol**	**Usual name of the protein subunit**	**Recommended name of the protein subunit**	**Site(s) of protein phosphorylation: serine/threonine kinase(s) involved**	**Effects of phosphorylation on the NADPH complex**
*CYBB*	gp91*^*phox*^* (also known as NOX2)	Cytochrome b-245 heavy chain	Ser486: Protein kinase C (PKC) isoforms	Phosphorylation promotes the catalytic activity and assembly of the NADPH oxidase complex
*CYBA*	p22*^*phox*^*	Cytochrome b-245 light chain	Thr147: Conventional PKC	Phosphorylation promotes NADPH oxidase complex assembly and activation.
*NCF1*	p47*^*phox*^*	Neutrophil cytosol factor 1	Between Ser303 and Ser379: PKC isoforms Ser304, Ser328: AKT Ser345: p38-MAPK, ERK1/2	Required for complex assembly and activation
*NCF4*	p40*^*phox*^*	Neutrophil cytosol factor 4	Thr154 and Ser315: PKC	Phosphorylation at Thr154 is required for NADPH oxidase complex assembly and activation at the phagosome
*NCF2*	p67*^*phox*^*	Neutrophil cytosol factor 2	Thr233: p38-MAPK, ERK1/2, PKC	Unknown

### MPO Release

In freshly isolated neutrophils from healthy subjects, fMLF also induces MPO exocytosis from primary granules (4, 12), which is an important part of the oxygen-dependent antibacterial arsenal (8, 12). Other toxic mediators stored in these primary granules are also released such as elastase and defensins. Stimulation of neutrophils triggers also the degranulation of two other cytosolic compartments, secondary and tertiary granules which contain common mediators (gelatinase, lysozyme, and ß2-microglobuline) and specific mediators, i.e., lactoferrin and acetyltransferase, respectively. The membrane of secondary and tertiary granules contains high amounts of gp91^*phox*^ (NOX2) and the fMLF receptor fPR1. Thus, during the process of MPO release by neutrophils stimulated with bacteria or bacterial peptides, the fusion of granular membranes to plasma membrane and phagosomes bring new pools of NOX2 and fPR1, which contribute to increase ROS production and bacterial killing. Experiments using selective pharmacological antagonists for phosphorylation of AKT, p38-MAPK and ERK1/2 showed that phosphorylation of AKT, p38-MAPK, but not that of ERK1/2, was involved in MPO exocytosis (4). Moreover, these pharmacological experiments have shown that p38-MAPK is activated downstream to AKT through mechanisms that remain elusive.

## Defective Response to FMLF in Neutrophils from Patients with Decompensated Cirrhosis

Freshly isolated blood neutrophils from patients with decompensated alcoholic cirrhosis stimulated *ex vivo* with fMLF exhibit a marked defect in ${\text{O}}_{2}^{-.}$ production (i.e., decreased NADPH oxidase activity), RB, and MPO exocytosis, and accordingly reduced bactericidal activity (3–5). These results confirm PN dysfunctions reported by other groups also showing impaired phagocytic activities (13–22). However, in some studies, basal ROS production by patient neutrophils was increased, despite defective bactericidal activity, which suggests a primed and or pre-activated state of patient neutrophils (17–19, 23, 24). We confirmed this paradoxical situation with some of our cirrhotic patients. However, the increased basal ROS production remained low which questions about its relevancy in antibacterial activity, in contrast to the high and rapid production of ROS induced by fMLF which leads to bacterial killing (4, 5). Because patients' neutrophils have decreased baseline protein expression of gp91^*phox*^, p22^*phox*^, and p47^*phox*^ (5), these decreased expressions likely contribute to defective fMLF-induced NADPH oxidase activation in these cells (summarized in the Figure 1B). The finding that here is no simultaneous decrease in the baseline mRNA levels of *CYBB* (encoding gp91^*phox*^, or NOX2), *CYBA* (encoding p22^*phox*^), and *NCF1* (encoding p47^*phox*^) in patients' neutrophils (5), suggests that decreased expression of the corresponding proteins is related to posttranscriptional mechanisms. Abnormally high plasma levels of elastase are found in patients with decompensated cirrhosis (25). Neutrophils from healthy subjects exposed to plasma from patients or purified elastase, but not neutrophils exposed to plasma from healthy subjects, exhibit a decrease in gp91^*phox*^ expression which can be prevented by the neutrophil elastase inhibitor, NEI) (5). These findings, together the finding that elastase released by fMLF-stimulated neutrophils from healthy subjects causes gp91^*phox*^ degradation (see above), suggest that high plasma elastase levels in patients may explain that their neutrophils have low baseline expression of neutrophil gp91^*phox*^ and p22^*phox*^. Since increased extracellular elastase levels do not affect p47^*phox*^ expression in neutrophils from healthy subjects (see above), high plasma elastase levels cannot explain decreased baseline expression of p47^*phox*^ in neutrophils from patients with decompensated cirrhosis. The mTOR protein complex 1 (known as mTORC1) promotes protein synthesis by regulating translation of several mRNAs into proteins (26) including gp91^*phox*^ (5). Because neutrophils from patients with decompensated cirrhosis exhibit decreased mTOR protein (5), this has been suggested to result in reduced translation of *NCF1* into p47^*phox*^ in these cells as well-translation of gp91^*phox*^ (5).

In addition to these alterations in protein expression of the NADPH oxidase complex, defects in signaling pathways have been shown in neutrophils from patients with decompensated alcoholic cirrhosis. Following fMLF stimulation, neutrophils from patients have decreased phosphorylation (i.e., activation) of AKTs, p38-MAPK, and ERK1/2 with no changes in the expression levels of corresponding unphosphorylated proteins and in the expression of the formyl peptide receptor [(3, 4), Figure 1B]. Together these findings suggest the existence of an important defect in signaling pathway, somewhere between the surface receptor and effector proteins. Alterations in the G proteins which are coupled to the formyl peptide receptor have been suggested to exist in neutrophils from patients with cirrhosis based on the impaired phospholipase C (PLC) activity in response to fMLF and fluoride, a G protein activator (16).

Neutrophil dysfunctions associated with cirrhosis are caused by intrinsic cellular alterations because they persist after cell washing (13). Neutrophils from healthy subjects exhibit decreased fMLF-induced MPO exocytosis when they are cultured with plasma from patients but not with plasma from healthy subjects (4), which reveals the presence of cell-permeant inhibitors in patients' plasma. Neutrophil dysfunctions have been shown to be reversible after removal of endotoxins (i.e., lipolysaccharide) from patients' plasma (17).

The defect in signaling pathways in patients' neutrophils may have several functional consequences. Decreased phosphorylation of AKT and p38-MAPK is associated with a defect in MPO exocytosis in patients' neutrophils (4) indicating a decrease in exocytosis of primary granules. In addition, activated AKT and p38-MAPK both phosphorylate a main component of NADPH oxidase, p47^*phox*^, at Ser304/Ser328 and Ser 345, respectively (Table 1). Therefore, defective activation of AKT and p38-MAPK may contribute to the defect in NADPH oxidase activity in patients' neutrophils. Finally, the defect in ERK1/2 phosphorylation may contribute to decrease NADPH oxidase activity, because ERK1/2 activation accounts for 30% of ROS production by fMLF-stimulated neutrophils from healthy subjects (4). Unlike ROS production, the MPO exocytosis induced by fMLF does not appear to be regulated by ERK1/2 (4, 27), but via the p38 MAPK, AKT (4), and PLC/calcium signaling pathways (12). These later are impaired in neutrophils from cirrhotic patients with may contribute to the defective MPO release (4, 16).

Interestingly, the defects in ${\text{O}}_{2}^{-.}$ production, MPO release, decreased phosphorylation of AKT, p38 MAPK, and bactericidal activity in patients' neutrophils can be reversed *ex vivo* by treatment of patients' neutrophils with toll-like receptor 7/8 (TLR7/8) agonists (4, 5). This treatment also stimulates a rapid *CYBB* transcription and translation into gp91^*phox*^ which is inhibited by rapamycin, indicating a mTOR-dependent process. These findings suggest that TLR7/8 agonists might be used in the future to restore neutrophil functions in patients with decompensated alcoholic cirrhosis. In support to this assumption, the TLR7/8 agonist R848 (Resiquimod) was recently shown to restore the impaired production of ROS in whole blood of cirrhotic rats and improve the survival of cirrhotic rats infected by bacteria (28). In this murine model of cirrhosis treated with R848, both neutrophils and mononuclear leukocytes (monocytes and lymphocytes) were modified as indicated by a decreased amount of these cells in the blood of ~40–50%, although modifications of their immune function remain not known.

In patients with decompensated cirrhosis, various defects have also been described in peripheral blood mononuclear cells (PBMCs), notably an impaired expression of genes induced by interferon type 1 (29), a decrease in antigenic presentation by lymphocytes and monocycte/dentritic cells and a decrease in lymphocyte proliferation (30). The effects of TLR7/8 agonists on these adaptive immunity cells in the context of cirrhosis are not known.

However, *in vitro* models, TLR7/8 agonists are particularly effective in inducing robust immune responses (31) including TNFα production in monocytes, IL12 production in human adult and newborn monocytes, INFα production in the dentridic cells, and the activation of T-regulatory cells. Because of their potent immunostimulatory properties, the TLR7/8 agonists are used in non-clinical and clinical studies as vaccine adjuvants (32). TLR 7/8 agonists are also evaluated for a variety of indications in clinical studies and animal models to treat various viral infections and skin cancer (31).

## Areas of Future Research

Studies should be performed in patients with cirrhosis to address several questions including the mechanisms for acquired defective signaling pathways in response to fMLF. In particular, the role of LPS should be investigated because it has been shown to be present in the plasma of patients with cirrhosis who did not have ongoing infection by Gram-negative bacteria (33) and because LPS may have effects on neutrophil ROS production (5). In addition, the responses to other potential stimuli for neutrophil activation should investigated in patients' neutrophils.

The phenomenon of neutrophil extracellular traps (NETs, which are mainly composed of DNA that is released from neutrophils upon pathogen encounter) has been considered an alternative to other nonlytic (apoptosis) or lytic (pyroptosis, necroptosis) cell death and called NETosis (6). NETs can entrap bacteria, fungi, and even viruses and contribute to antimicrobial defense. NETosis relies on the presence of the major neutrophil serine protease elastase, MPO and active NADPH oxidase (8). Therefore, NETosis would not be expected to occur in neutrophils from patients with cirrhosis which have defect in NADPH oxidase and in MPO exocytosis. Because defective NETosis might contribute to the fact that patients' neutrophils have decreased ability to kill bacteria, NETosis should be investigated in these cells.

It will be also important to investigate, in patients with cirrhosis, the phenotype of circulating neutrophils according to the severity of the disease identifying cell-surface markers and transcriptome profile, and investigating their ability to leave circulation toward tissues.

Finally, the efficacy and safety of TLR7/8 agonists should be further investigated *in vivo* preclinical experiments performed in mouse models of chronic liver disease.

## Conclusions

Neutrophils from patients with decompensated cirrhosis exhibit marked alterations that may explain the susceptibility of these patients to develop bacterial infections. These neutrophil alterations include marked defects in fMLF-induced activity of the NADPH oxidase complex, MPO release, and bactericidal activity. Decreased activity of the NADPH oxidase complex is related to reduced levels of expression of its major components through post-transcriptional mechanisms. A defect in the AKT/p38 MAPK signaling pathway may explain the decrease in phosphorylation of p47^*phox*^ (an important component of the NADPH oxidase complex) and MPO release, in response to fMLF stimulation. Most of these alterations are reversible *ex vivo* with TLR7/8 agonists.

## Author Contributions

RM and AP wrote the manuscript. AP and VA provided critical revision of the manuscript for important intellectual content. AP drew the figures.

### Conflict of Interest Statement

The authors declare that the research was conducted in the absence of any commercial or financial relationships that could be construed as a potential conflict of interest.
